# Dental Students

**Published:** 1879-12

**Authors:** D. C. Hawxhurst


### DENTAL STUDENTS.

BY D. C. HAWXHURST.

I have been asked so many times to take a dental student, that I am growing sensitive over the subject. If I see any old lady coming up my stairway now-a-days with her hopeful young son of fourteen (who has perhaps proved a failure at all kinds of work and has run away from school) I always tell her "no!" before she gets fairly into my reception room. I can tell a proposed dental student before he gets there. I

have had all the dental students I want. Many people in this country think that if a boy is so unsteady that he can not be put to a trade, and has so much mental inability that he is good for nothing in medicine, law or divinity, he may still make a tolerable dentist. And, indeed why should not one who believes that it is the whole duty of a dentist to tinker teeth and charge large bills for the same, regard such a boy as fit for the position? At any rate, such boys are often brought to me in the hope that I can make something of them. I have the misfortune to be regarded by some of these people as a model of morality, and they probably think my influence would be good over the boy, entirely forgetful of what his influence might be on me.

Such boys have often proved to be worthless and a bill of expense to their parents, who now desire that they should "earn something from the first." Their expectations are usually modest; they only wish that the boy should be "self-supporting." When they ask me for my terms, I usually tell them that I think I could afford to have such a boy about my office during business hours for about a dollar a day. This ends the interview.

Another class of applicants for a dental pupilage, may be fitly represented by a Mr. Z., who made his appearance some time since with testimonials of moral character, and certificates from his high school teacher, claiming that he could "run a laboratory alone, fill teeth or anything." He would like "to place himself under my distinguished instruction for a period." Having much to do I hired him for a few weeks, during which time I learned the following particulars: He had been under the instruction of two of the worst quacks in my native town, one of them during the period of three months, the other six weeks. He desired to spend a few months with me, until he could open an office for himself. When I learned that he could not spell; knew nothing of the use of capital letters; had no idea of grammar and was as ignorant of English literature as if it had been written in the Choctaw tongue; that he would lie and wear soiled linen; that he hated tobacco, but chewed fine cut in secret and smoked cigars clandestinely as soon as I had made a first payment to him; that he drank beer and was interested in the ladies; that he persisted in washing with soap, but always forgot to empty the bowl; got down a display of books which he never studied and never returned to their places, and that he even usurped my place in the operating room, mutilating innocent jaws while I was at dinner—when I learned these thing I told him one day that I wanted to see him alone, I desired to talk with him about something.

Passing over other things I shall give a brief hint of what I advised him touching his education. First I told him that he should begin in a common school and study reading, spelling, writing and arithmetic, making a special effort to learn the English language. I had previously ascertained from him that he knew no other and that he expected to practice in an English speaking country, viz. Michigan. I assured him that the better class of the community would certainly be astonished and shocked at any written communication which he might have occasion to send to them. I advised him to study at least through our Union School and then if possible go to college, if not to graduate at least to take some special studies there for a year or two. I advised him to go then to some one of the better class of dental colleges and stay two or three years.

When I remark that this young gentleman was a handsome man, you will not be surprised that he resented my strictures on his education. He was inclined to defend himself and thought that I underrated a large amount of school education which he posessed, though he "had no capacity to show it off to advantage."

I have had a good many applications from those who would become pupils. They are generally poor and wish to make a humble requisition of about five dollars a week for expenses [illegible]m the very start. They must live as they go and they generally "know a great deal about the mechanical part of dentistry." They believe they can do the whole of my work without any aid from me.

The truth is that any one of these boys would occasionally be able to make a fair set of teeth that could be worn by the patient, especially if she lived a long way off in the edge of the uncleared frontier forests. He would meet with such a success as this in about one case out of every ten. In the other nine cases, the blocks would come out of the flask broken or displaced, the plate would be badly warped or the celluloid would be entirely scorched away. Such a boy would usually invent many new and unknown varieties of destructive contingency. I have estimated that it will generally take about three months for such a boy to drive a man's business all from him and do away with the necessity for for both laboratory and boy. In less time than three months some of his patients will not have heard that he is having "bad luck with every set of teeth" and thus he may have isolated applications even after it has become generally understood that he is " better in the operative department, than in the mechanical."

As a general rule the worst piece of furniture that a dental practitioner can put into his office is a dental student. For assistance, it is better to rely upon some faithful young lady who will be agreeable to patients and who will serve the interests of her employer better than three dental students and for less money than one of them will demand.

In a future article I shall say something about dental students from their standpoint. If I have in this article seemed to make out a case against the dental student, I shall no doubt in that find something good to say of him and may possibly make out something of a case against his preceptor.

